# Stereotactic arrhythmia radioablation (STAR) opens a new era in the treatment of arrhythmias?

**DOI:** 10.3389/fcvm.2024.1449028

**Published:** 2024-09-27

**Authors:** Shuang Zhang, Lin Hu, Hanze Tang, Liyi Liao, Xuping Li

**Affiliations:** Department of Cardiovascular Medicine, The Second Xiangya Hospital, Central South University, Changsha, Hunan, China

**Keywords:** stereotactic arrhythmia radioablation (STAR), arrhythmia, ventricular arrhythmia, atrial fibrillation (AF), noninvasive treatment

## Abstract

Tachyarrhythmias are common cardiovascular emergencies encountered in clinical practice. Among these, atrial fibrillation (AF) and ventricular tachycardia (VT) pose significant hazards due to their prevalence and severity. Initially, non-invasive pharmacological antiarrhythmic interventions were the primary treatment modality; however, due to their limited control rates and side effects, invasive therapies have been introduced in recent years. These include catheter ablation, alcohol ablation, cardiac implantable electronic devices, and heart transplantation. Nonetheless, for some patients, invasive treatments do not offer a definitive cure for arrhythmias and carry the risk of recurrence, especially with AF and VT, where the relapse rates are high and the treatment for VT is correlated with the type of tachycardia present. Currently, novel non-invasive treatment methods are emerging, with stereotactic radioablation therapy becoming an effective alternative for the management of refractory tachyarrhythmias. This review provides an overview of the application background of Stereotactic Arrhythmia Radioablation (STAR) therapy and promising results from its use in animal models and clinical applications.

## Introduction

Cardiovascular diseases are the leading cause of death globally. It is estimated that about 17.9 million people die from cardiovascular diseases each year, accounting for 31% of all global deaths (1). Among these, sudden cardiac death caused by arrhythmias, such as ventricular arrhythmias (VA) or atrial fibrillation (AF), constitutes 15%–20% of all deaths (2).

VA are often closely associated with structural heart diseases caused by ischemic cardiomyopathy (ICM) or non-ischemic cardiomyopathy (NICM), as well as related to ion channelopathies, and can even occur in hearts with normal structure. Among these, Monomorphic Ventricular Tachycardia (VT) is the most common type of VA (3). The most severe consequence of VT is Sudden Cardiac Death (SCD). According to data from the American Heart Association, there are over 550,000 cases of cardiac arrest each year, accounting for more than half of all cardiovascular disease-related deaths (4). In patients with structural heart disease, the use of antiarrhythmic drugs such as amiodarone is often indicated to manage VT. Amiodarone is preferred due to its efficacy in reducing VT episodes, especially in patients with both ischemic and non-ischemic cardiomyopathy. VT ablation is typically indicated in cases where VT is refractory to medical therapy, where there is a high burden of VT, or where VT is causing hemodynamic instability. ICM, often resulting from coronary artery disease, presents distinct electrophysiological challenges compared to NICM, which can arise from a variety of etiologies including dilated cardiomyopathy, hypertrophic cardiomyopathy, and myocarditis. Ablation strategies and outcomes can vary significantly between these two types of cardiomyopathy, necessitating a tailored approach to each patient.

In ICM, VT typically originates from subendocardial scars, which are more accessible for catheter ablation *via* the endocardial surface. In contrast, VT in NICM often originates from intramural or epicardial foci, making it harder to reach through endocardial ablation alone, resulting in lower success rates. Research suggests that combining endocardial and epicardial ablation significantly reduces VT recurrence risk (5). However, epicardial puncture carries higher risks and surgical complications (6).

Some studies indicate that short-term success rates for VT ablation are similar in both NIDCM and ICM patients, but long-term outcomes are notably worse for NIDCM patients (7), though NIDCM representation was low in these studies. Another study with a higher proportion of NIDCM patients demonstrated significantly lower success rates for these individuals (8). A meta-analysis further confirmed the higher risk of VT recurrence in patients with non-ischemic dilated cardiomyopathy and arrhythmogenic right ventricular cardiomyopathy (5).

This discrepancy underscores the clinical need for alternative therapies like stereotactic arrhythmia radioablation (STAR), especially in NICM patients where conventional ablation may be less effective. While combined endocardial and epicardial ablation can improve outcomes, the associated surgical risks highlight the potential of STAR as a less invasive alternative with fewer complications.

According to research reports, treatment with amiodarone can reduce the recurrence rate of ventricular tachycardia by 71% within the first year (9). However, when undergoing long-term treatment, it is crucial to be vigilant about the significant risk of associated side effects (10, 11). When patients have a poor response to drug therapy, Catheter Ablation (CA) can serve as a preferred treatment option. Multicenter clinical studies have shown that, in both ICM (8) and NICM (12, 13), although CA does not reduce mortality in VT patients, it does reduce the composite endpoint of cardiovascular death, Implantable Cardioverter Defibrillator (ICD) shocks, hospitalizations due to heart failure, or severe treatment-related complications compared to antiarrhythmic drugs (AAD). While CA has seen significant improvements in efficacy, about 50% of patients still experience recurrence after the first VT ablation and may require multiple CA treatments. In some cases, for example, patients with left ventricular dysfunction (left ventricular ejection fraction ≤35%) following a myocardial infarction, or those with certain genetic heart diseases or syndromes (such as Long QT syndrome, Brugada syndrome, or hypertrophic cardiomyopathy), the consideration of an ICD may be necessary. ICDs can automatically detect and correct malignant ventricular arrhythmias, playing a crucial role in managing patients at high risk of SCD. Although ICDs can effectively terminate ventricular tachycardia, recurrent arrhythmias and the shock therapy from ICDs may reduce the patient’s quality of life and may be associated with increased mortality, heart failure, and higher risk of hospitalization. Therefore, a comprehensive treatment usually also involves AAD. The current focus of new treatment methods is on neuromodulation therapies, especially in the field of polymorphic VT, Ventricular fibrillation (VF), and scar-related monomorphic VT. Common techniques include single-shot percutaneous stellate ganglion block (14, 15), continuous stellate ganglion block (16), and cardiac sympathetic denervation (17, 18). These therapies offer protection against arrhythmias, potentially slow the VT cycle length, and aid in mapping and subsequent ablation.

Similar to ventricular arrhythmias, AF also holds a pivotal position among arrhythmias. Epidemiological studies show that atrial fibrillation significantly increases the risk of all-cause mortality and stroke (19), and imposes a severe economic burden. Currently, over 33 million people worldwide are diagnosed with atrial fibrillation (20), and both its incidence and prevalence are closely related to aging. Data indicates that in 2017, there were 3.046 million new cases of atrial fibrillation globally. The estimated incidence rate in 2017 (403 per million residents) was 31% higher than the corresponding rate in 1997. The global prevalence of atrial fibrillation was 375.74 million cases (accounting for 0.51% of the global population), which also increased by 33% over the past 20 years. Future projections suggest that by 2050, the absolute burden of atrial fibrillation could increase by more than 60% (21).Similar to ventricular arrhythmias, in patients with atrial fibrillation, although CA and AAD are cornerstones in the treatment of arrhythmias, the conversion rate for drug therapy is below 50%. AF ablation is indicated not only after the failure of antiarrhythmic drugs but also as a first-line treatment for specific patient populations. Current guidelines suggest considering catheter ablation as an initial therapy in patients with symptomatic paroxysmal AF, particularly when there is a desire to avoid antiarrhythmic drug therapy, or when there are contraindications to such drugs. The decision to pursue ablation as a first-line treatment is based on factors including patient preference, AF symptom burden, and the presence of underlying heart disease. The success rate and risk of complications from ablation vary depending on the type of procedure and are largely dependent on the atrial fibrillation substrate, with success rates of 70%–80%. Therefore, some patients may require multiple catheter ablation procedures. In addition to traditional radiofrequency ablation, emerging modalities such as cryoablation (22), pulse field ablation (23–25), laser ablation (26), high-intensity focused ultrasound ablation (27), shock wave ablation (28, 29), and chemical ablation with ethanol (30–32) are showing promise in the treatment of both ventricular and atrial arrhythmias.

In recent years, significant advancements have been made in the treatment of arrhythmias through the development of new non-invasive ablation technologies. Stereotactic body radiation therapy (SBRT), also known as STAR, offers a new treatment option for ventricular tachycardia and atrial arrhythmias in patients who respond poorly to drug therapy. This treatment not only provides a safer solution with a higher success rate but also has lower costs compared to traditional therapies. Recognized by recent American and European guidelines, the neuromodulation therapies are well-established and can be used alongside STAR to optimize patient outcomes. Stellate ganglion block, in particular, can act as a bridging therapy to VT ablation, addressing interim arrhythmia control during the planning phase of STAR.

## Literature search method

The literature search was conducted using multiple databases, including PubMed, Scopus, and Web of Science, with search terms such as “STAR therapy,” “ventricular arrhythmias,” “stereotactic body radiation therapy,” and “cardiac radioablation.” We included studies published in English from 2000 onwards.

The selection criteria for including manuscripts in this review were based on relevance to the topic, quality of the study design, and the significance of the findings related to the use of STAR in treating arrhythmias. We prioritized peer-reviewed articles, clinical trials, and consensus guidelines. Articles were excluded if they were case reports with insufficient data, non-peer-reviewed, or focused on unrelated aspects of cardiac arrhythmias.

## Preclinical studies on STAR

Early preclinical studies on stereotactic radiosurgery treatment, specifically experiments on animal models, can be traced back to 2010. At that time, researchers like Sharma A (33) conducted targeted studies on key conduction areas of the heart, such as the tricuspid valve isthmus, the atrioventricular node, the pulmonary vein-left atrial junction, and the left atrial appendage. They proposed a hypothesis that radiation-induced fibrosis could be used to modulate the heart’s electrical conduction pathways through radiation therapy. The experimental results demonstrated that a radiation dose of 25 Gy was sufficient to effectively produce electrophysiological changes, and this effect lasted for at least 90 days. Particularly at the pulmonary vein-left atrial junction and the left atrial appendage area, a significant reduction in voltage to below 0.05 mV was observed, with no spontaneous arrhythmias detected. This technique achieved effective bidirectional conduction block at the tricuspid isthmus and the atrioventricular node, while no radiation damage was found outside the target areas, and histological samples also displayed the anticipated effects of x-ray radiation.

However, subsequent researchers, including Blabck O et al. (34), reached conclusions that differed from previous findings: a single dose of 25 Gy was not sufficient to produce transmural radiation-induced fibrosis, and such full-thickness myocardial fibrosis could only be induced in animal models with a single dose significantly exceeding 30 Gy. This discovery aligns with the conclusions reached by Sharma A and others in their research, indicating that a single high dose of radiation, ranging from 40–60 Gy, could induce myocardial fibrosis and block cardiac conduction.

Based on this research, some scholars have suggested that the biological effects caused by radiation might be related to the regulation of myocardial electrical conduction. Studies by Zhang DM et al. (35) on mouse models indicate that radiation effects could involve changes in gap junctions and their constituent proteins (i.e., connexins), leading to alterations in conduction velocity and refractory periods. In their experiments, the researchers explored the specific impacts of ionizing radiation on cardiac function. Consistent with previous studies, they did not observe sufficient CA-like scar formation in clinical samples of cardiac patients treated with 25 Gy of photon radiotherapy. In mouse hearts, a radiation dose of 25 Gy was found to sustainably increase the expression levels of cardiac conduction proteins, thereby enhancing ventricular conduction function. This effect is believed to be due to the increased expression of the Na V 1.5 ion channel and Cx43 gap junction protein.

## Clinical data on STAR

Our understanding of the potential radiogenic damage caused by STAR to the heart as a whole or to its individual substructures, both in the short and long term, is still quite limited. Similarly, the biological response of cardiac tissue to radiation, whether in normal or pathological states, has not been fully elucidated. Clinically, data on this therapy primarily come from the treatment of VT, with clinical trials by Cuculich PS (36) and others demonstrating that after treatment with 25 Gy, the VT burden was reduced by 99.9%. Another prospective phase I/II clinical trial targeting refractory ventricular tachycardia indicated (37) that patients treated with 25 Gy experienced a 94% reduction in VT episodes or ventricular premature contractions (PVCs). In 89% of these patients, the frequency of VT episodes or PVC burden decreased by more than 75%. The overall survival rate was 89% at six months post-treatment and 72% at twelve months. In these cases, therapeutic effects were often observed within days to weeks, much earlier than the expected timeframe for radiation-induced fibrosis to occur. After conducting autopsies on patients who had undergone Stereotactic Body Radiation Therapy (SBRT), Krug D (38) and other scholars found that transmural radiation of 25 Gy did not result in noticeable local fibrosis in the target area. This is consistent with previous preclinical study results, which indicated that a dose exceeding 40 Gy is required to produce lesions sufficient to disrupt conduction.

Currently, reports on the application of STAR for patients with refractory VT are mostly derived from small-scale, single-center experiences sharing various methods and techniques, with relatively short follow-up periods. Moreover, the number of patients undergoing this treatment is relatively low across Europe and globally. Within the STOPSTORM project, funded by a multidisciplinary alliance (39), a retrospective study was conducted. This study aimed to establish a platform named “Standardized Treatment and Outcomes Platform for Recurrent Tachycardia Stereotactic Therapy” as a network foundation for extensive research on STAR. This platform is dedicated to integrating treatment data, evaluating the application patterns and therapeutic effects of STAR, and promoting the standardization of STAR implementation across Europe. Although most patients received a single dose treatment of 25 Gy, specific planning techniques and dosage prescriptions still vary in practical application.

Some scholars believe that the treatment of AF with STAR has broad development prospects and may surpass current AF treatment methods in terms of cost-effectiveness. The first application of STAR in pulmonary vein isolation treatment for AF was reported by Qian et al. (40) in 2019, which described two cases of paroxysmal AF patients who had not previously undergone invasive catheter ablation. They received 25 Gy of radiation treatment. Using real-time imaging with planar x-ray technology, the research team located the treatment area near the left atrial base adjacent to the right atrial septum by inserting a marker catheter *via* the vein, and based on this, they developed a treatment plan, conducting a radiation therapy session lasting about 90 min. Cardiac magnetic resonance imaging confirmed atrial fibrosis caused by the radiation treatment. At a six-month follow-up, one patient experienced a recurrence of AF; the other patient did not exhibit any arrhythmic events during a two-year follow-up period. However, due to the limited sample size of this study, its results cannot serve as strong evidence for widespread application.

At the same time, a team led by Professor Yang Qing from West China Hospital of Sichuan University (41), successfully treated a patient with refractory atrial tachycardia using STAR technology in 2022. With the aid of a three-dimensional electrocardiogram system, the activation map showed that the origin of the atrial tachycardia was located near the right coronary artery on the epicardial surface of the right atrium. To avoid damaging the right coronary artery during the ablation process, which could cause acute myocardial infarction, the medical team opted for the STAR technique for treatment. The 11-month follow-up after the treatment showed no recurrence of atrial tachycardia in the patient. This case represents an advanced application of STAR technology in the treatment of atrial arrhythmias.

## The origin of STAR

Traditional radiation therapy (RT) works by applying high doses of radiation directly to cells, exerting toxicity that inhibits their division, thereby inducing damage to the target tissue. The most common source of radiation is medical linear accelerators (LINACs), which can produce photon or electron beams. In addition, there are systems like the Gamma Knife, which utilizes cobalt-60 radioactive isotopes to generate gamma rays, as well as various large particle accelerators, such as proton or heavy ion (such as carbon ion) accelerators, with photon radiation therapy being the most common.

External beam radiation therapy refers to the use of high-energy photons generated by medical LINACs (Linear Accelerators) to irradiate patients from outside the body. Stereotactic body radiotherapy (SBRT) employs traditional LINACs or robot-assisted LINACs (such as CyberKnife) for indirect ionizing photon radiation therapy, combining the high precision of LINACs, image-guidance systems, and robotic technology. Compared to traditional linear accelerator radiation therapy, SBRT utilizes three-dimensional imaging to precisely locate lesions and irradiates the target area from multiple angles. This achieves concentrated irradiation of the lesion with high-energy, high-dose radiation from outside the body in a non-invasive manner, precisely destroying the diseased tissue while minimizing damage to the surrounding healthy tissue.

Stereotactic radiosurgery (SRS) is a highly precise form of single-session radiation therapy that, unlike traditional radiation therapy which disrupts the cell division mechanism, concentrates high doses of ionizing radiation on a small area of the body to induce local necrosis. This technique was introduced by Swedish neurosurgeon Lars Leksell (42) in 1951, initially for the treatment of intracranial tumors, with the aim of delivering high-dose treatment to the target tissue while minimizing damage to surrounding normal structures. With continuous advancements in radiation therapy technologies, such as imaging, tracking, and intensity modulation, the application of SRS has expanded to the treatment of various tumors in the head and neck, lung, abdomen, pelvis, and spine, demonstrating excellent local control rates and lower side effects.

STAR represents an emerging application of SRS in the field of non-invasive treatment for arrhythmias. It minimizes the risks associated with vascular interventions and other surgical risks (43), offering an innovative approach to the treatment of arrhythmias.

## Mechanism of STAR

Traditional radiation therapy (RT) delivers high doses of radiation to target tissues in a non-invasive manner. When x-ray beams are emitted from a LINAC, a multitude of photons with varying energies (4–20 MeV) undergo several types of interactions within the medium. The type and probability of these interactions depend on the incident photon’s energy and the atomic number of the medium. The four main interactions that occur within the medium include the photoelectric effect, Rayleigh scattering, Compton effect, and pair production. The ionizing radiation produced after these interactions (44) can cause direct or indirect damage to DNA. Direct damage involves the ionization or excitation of electrons within DNA atoms, which can disrupt chemical bonds, leading to DNA strand breaks or base damage. Indirect damage involves free radicals generated by radiation interacting with the medium surrounding DNA, causing single and double-strand breaks in DNA. During attempts by the cell to replicate, such DNA damage can lead to cell death during the mitotic process, a mechanism widely applied in the treatment of malignant tumors.

Normal cells have a stronger DNA damage repair capability than cancer cells and can complete self-repair between a single radiation treatment or multiple radiation cycles. This results in differential effects of radiation on cancer cells and normal tissues. This type of cell death may take several weeks to fully occur.

The STAR technique precisely locates ionizing radiation to specific areas of the heart based on preoperative imaging and non-invasive electrocardiograms during arrhythmia episodes. Since the target myocardium lacks rapidly dividing cells, the direct damage from ionizing radiation is relatively minor. Another possible mechanism of action is the induction of tissue hypoxia or necrosis through cell apoptosis and microvascular damage, leading to the formation of fibrosis and scars, thereby isolating abnormal circuits or eliminating the initiation points of arrhythmias (45). In STAR treatment, ionizing radiation may also cause significant vascular damage and may alter the microenvironment of cardiomyocytes, thus disrupting the substrate for rapid arrhythmias.

While some studies have summarized radiation-induced changes in tumor vasculature (44), the vasculature in xenograft models, being of host mouse origin, may not fully apply to the vascular changes that might occur in human tumors. Extensive research indicates that tumor vessels experience moderate damage under a single radiation exposure of 5–10 Gy, whereas radiation per fraction exceeding 10 Gy causes severe and lasting vascular occlusion. In contrast, normal tissue vasculature exhibits significant radioresistance. Unlike tumor vessels, normal vessels are composed of continuous endothelial cells closely supported by basement membranes and pericytes, with a relatively intact structure. Soon after irradiating rats with a single dose of 10–60 Gy, Song CW (46) and others observed that blood vessels in the skin and muscle significantly dilated, with an increase in blood flow, and this dilated state could be maintained for more than 12 days. Therefore, normal tissues typically exhibit endothelial cell damage months or years after high-dose irradiation, leading to vascular fibrosis.

Despite this, electrophysiological effects and fibrosis typically begin to manifest 6–8 weeks after irradiation, with experiments showing that at least 25 Gy (33), or higher doses of radiation are necessary to cause damage sufficient to alter electrophysiological properties. However, subsequent studies have refuted the notion that fibrosis occurs with just a 25 Gy dose, concluding that transmural radiation-induced fibrosis can only be induced in animal models with a single dose significantly exceeding 30 Gy (34). This aligns with the findings of Sharma A et al. (33), indicating that a single high dose of 40–60 Gy of radiation can induce myocardial fibrosis and conduction block.

Building upon this research foundation, scholars (47) conducted in-depth immunological analyses of tissues after radiofrequency ablation. The analysis revealed long-term vascular damage, fibrosis, loss of function and polarity of cardiomyocytes and Purkinje fibers, accompanied by vacuolization in the tissues subjected to radiofrequency ablation. These structural and functional changes in tissues further triggered physiological alterations in the heart, primarily attributed to radiation-induced fibrosis and inactivation of myocardial cell function. However, the full manifestation of fibrotic responses is expected to take several months to years.

Similarly, the findings of Krug D et al. (38) align with previous preclinical studies, indicating that doses exceeding 40 Gy are required to induce lesions sufficient to interrupt conduction. Moreover, in clinical practice, the reduction in ventricular tachycardia (VT) typically occurs within weeks following radiation therapy at 25 Gy, a time frame in which radiation-induced fibrosis alone cannot fully account for the breadth and speed of these effects. Therefore, the current understanding of radiation-induced fibrosis does not fully explain the rapidity and magnitude of VT reduction observed clinically.

Another novel hypothesis is that radiation-induced effects may involve changes in gap junctions and their structural proteins, leading to alterations in cardiac conduction velocity and refractory period (35). In addition to mouse experiments, the study also observed 19 patients undergoing radiation therapy, finding no significant statistical differences in their QRS intervals, but a trend towards QRS shortening. Meanwhile, when irradiating the heart after myocardial infarction, the experiment found that protein expression and electrophysiological effects were limited to functional myocardium, with no such effects observed in scar tissue.

This finding is similar to the results of Gianni C et al. (48), who studied 5 patients with refractory scar-related VT. The results indicate that although the STAR method did not cause significant side effects, its long-term efficacy in controlling arrhythmias in high-risk populations, especially those with scar-related ventricular tachycardia, was not remarkable. This indirectly suggests that the mechanism of action of SBRT does not appear to involve tissue fibrosis caused by cell growth inhibition or cytotoxic effects, but rather through modulation of myocardial conduction.

Different studies have shown varying conclusions regarding different radiation doses. Immediate changes may be related to the heterogeneity of functional myocardial structural proteins and electrical conduction, while the progression of fibrosis varies depending on the target tissue (atrial working myocardium, nodal and conduction tissue, ventricular working myocardium), with the dose required to induce fibrosis potentially differing for each.

## The operational procedure and energy selection for treatment

Traditional radiation therapy typically divides the total dose into small treatment fractions over consecutive weeks, while SBRT utilizes a series of high-energy non-coplanar beams to focus concentrated high-dose radiation on a single target volume. These beams can precisely irradiate the target area from various angles, significantly reducing damage to surrounding healthy tissues. Given SBRT’s characteristic of delivering high doses to small target volumes with rapid dose falloff at the edges, precise delineation of target boundaries and accurate positioning become crucial. The efficacy of SBRT relies heavily on the precise application of IGRT techniques and stringent QA measures in advanced dose calculation. Prior to treatment, the physics team should conduct comprehensive end-to-end testing to ensure adherence to the intended design of STAR therapy. Each step of the treatment process, including imaging modalities, positioning devices, simulation procedures, respiratory and cardiac motion management, treatment planning, and implementation, requires meticulous testing. Successful implementation of STAR ablation necessitates close collaboration among radiation oncologists, medical physicists, radiation oncologists, cardiologists, cardiac electrophysiologists, and other healthcare professionals. The involvement of a multidisciplinary team is crucial for patient selection and optimization of STAR planning and treatment.

**Figure 1:**
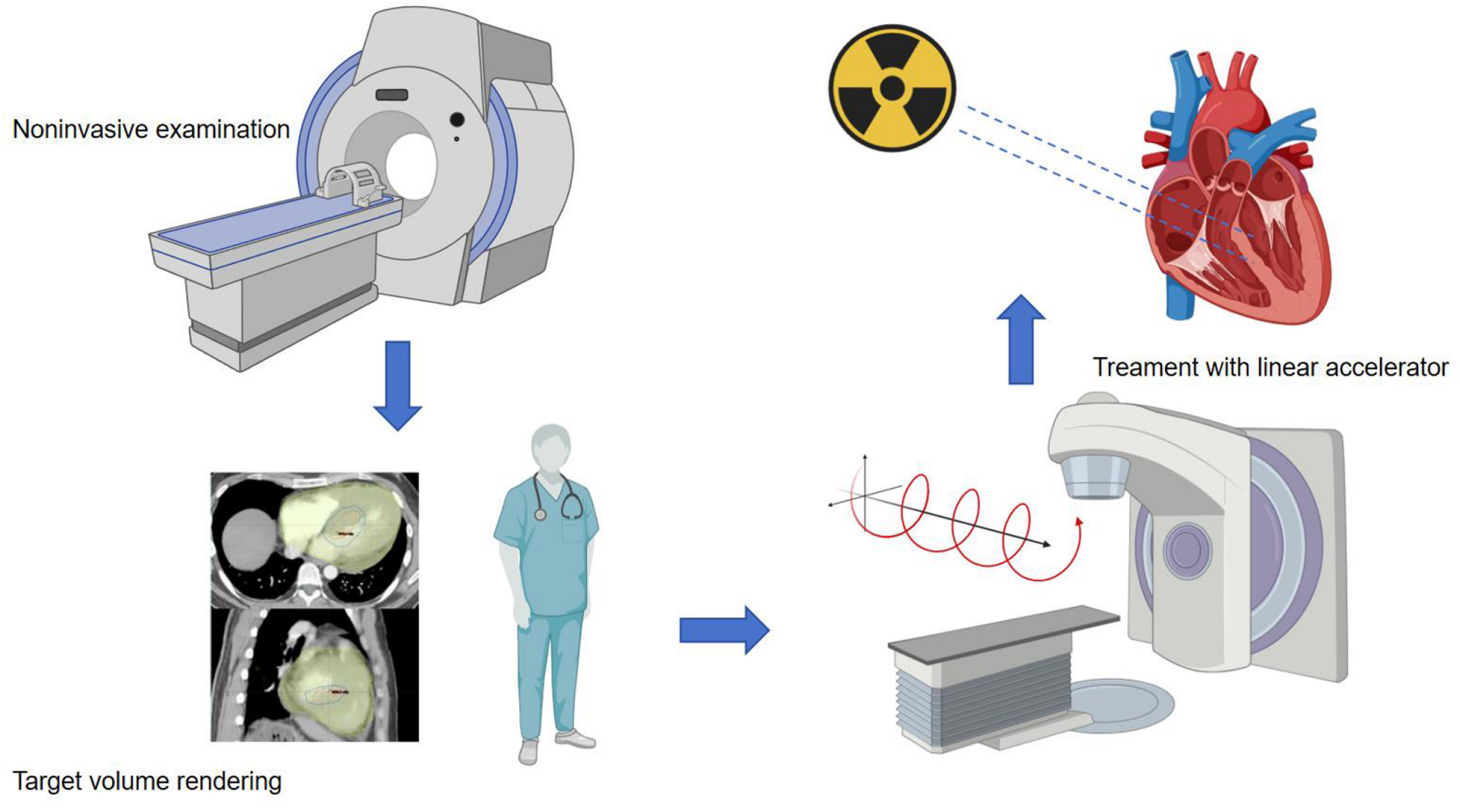
Procedure workffow. The main source of data used to identify the target for STAR is the electroanatomical map of the last VT ablation, eventually integrated with non-invasive data. When invasive data is not available at all, the patient undergoes non-invasive imaging examinations such as CT, echocardiography, and cardiovascular magnetic resonance imaging (CMR). Arrhythmia specialists use the anatomical information obtained from these imaging processes to determine the radiation volume. Radiation oncologist and the electrophysiologist transfer the target volume to a respiration-correlated four-dimensional CT, allowing for the assessment of overall cardiac and pulmonary motion. The patient is immobilized using a vacuum-assisted device, and stereotactic radiotherapy is administered through image-guided radiation therapy equipment with a linear accelerator. The radiation therapy is accurately targeted at the specified objective.

In the preclinical phase, for patients about to undergo stereotactic ablation therapy, the first step is to establish an ablation plan. Radiation therapy planning typically involves collaboration with the electrophysiology team to obtain electroanatomic maps of arrhythmias invasively or non-invasively. Ideally, when there is a single morphology of arrhythmia present, accurate localization is crucial for treatment planning. Non-invasive cardiac imaging modalities, such as echocardiography, computed tomography (CT), magnetic resonance imaging (MRI), or positron emission tomography (PET), are highly valuable for identifying and locating myocardial scar areas that may cause arrhythmias. Additionally, non-invasive mapping systems based on electrocardiographic imaging (ECGi) (49) provide an effective auxiliary method for mapping VT, identifying exit sites, and targeting VT treatment with STAR, thereby enhancing the precision of ablation procedures. After obtaining imaging data of these arrhythmia substrates, combined with non-invasive ablation techniques, radiation oncologists and the electrophysiologist then delineate the target volume (TV) for treatment and formulate specific treatment plans. The flowchart is as follows (Figure 1).

To date, STAR therapy for refractory VT patients has demonstrated high efficacy in reducing VT burden and minimizing interventions with ICDs, with fewer side effects. Combining the aforementioned research findings, it suggests that even at lower doses (20–25 Gy), STAR can rapidly affect the cardiac conduction system, while at higher doses (exceeding 30 Gy), it may lead to scar tissue formation. This difference in mechanism poses further challenges for precise dose prescription, uniformity of dose distribution, and determining maximum doses.

Despite its complexity, preliminary clinical data for STAR has shown promising results. Significant relief in VT burden was observed following treatment with a 25 Gy dose. The first prospective clinical trial assessing the safety and efficacy of STAR, conducted by Robinson et al. (37), reported low acute toxicity, comparable one-year survival rates to similar patients, and substantial improvement in patients’ quality of life (QoL) over time, with a significant reduction in VT burden.

In addition to photons, particle radiation also includes electrons, protons, and heavy ions (such as carbon ions). Electron beams release energy at shallow depths and are typically used to treat tumors near the skin surface.

Protons release energy steadily as they penetrate tissue, but at a specific depth, known as the Bragg peak, they release the maximum energy abruptly and then quickly stop (50). This characteristic allows proton radiation to concentrate high doses in the tumor area while minimizing impact on surrounding healthy tissue. The first case (51) using proton STAR for the treatment of refractory VT achieved promising results, with no VT episodes for two months post-procedure. Although 11 VT episodes occurred on days 54–59, they were slower and hemodynamically stable. While proton STAR therapy shows reduced toxicity to surrounding tissues, further research is needed to precisely target VT sites, optimize compensation for cardiopulmonary motion, determine the most appropriate proton dose, and follow up for long-term outcomes.

Similar to protons, heavy ions (52) also exhibit the Bragg peak effect, but their energy release is more concentrated, and their biological effects are stronger. Heavy ions have a more significant biological effect than protons and photons, causing more pronounced cellular damage and greater DNA damage, making them more effective for treating certain resistant or highly refractory tumors. Protons and heavy ions can focus most of their energy on the tumor site, thereby reducing damage to surrounding healthy tissues. In contrast, photon and electron radiation therapy release more energy along the path as they enter the body, leading to more widespread tissue damage. The latest research developments have introduced protons (53) and heavy ions (54) into the treatment of ventricular arrhythmias. Animal models have confirmed their safety and efficacy, but further clinical studies are needed (55). And proton therapy can cause some damage to Cardiac implantable electronic devices (CIED) (Reprogrammable CIED reset and battery depletion) (56), so more precise patient selection may be necessary.

## Limitations

From the above discussion, it is evident that the success of SBRT heavily relies on the integration of IGRT technology and advanced dose QA procedures. Due to the continuous motion of the heart and the physiological motion related to respiration affecting cardiac structures, motion tracking and precise fixation are crucial for SBRT targeting myocardial tissue. Additionally, other structures near the heart, such as heart valves, coronary arteries, esophagus, phrenic nerve, lung parenchyma, pericardium, and atrial tissues, are also at risk of radiation-induced injury and are similarly in motion. Considering the complexity of thoracic anatomy, the treatment system must be highly accurate and capable of real-time tracking of moving targets. Organ motion from free breathing and the heartbeat itself pose additional challenges related to target accuracy, necessitating the use of dynamic modeling with clinically relevant motion parameters (such as amplitude and cycle time) for end-to-end testing to assess the precision of the entire treatment chain (including CT simulation, treatment planning, and delivery).

In clinical research, Neuwirth et al. (57) reported a case series study of 10 patients with NYHA class II or III heart failure symptoms and refractory scar-related VT, primarily caused by ischemic heart disease, who underwent ICD implantation. These patients received radiation ablation therapy at a dose of 25 Gy, with 8 patients experiencing VT recurrence after 90 days and 3 patients experiencing electrical storms. Similarly, Gianni et al. (48) reported 5 patients who experienced clinically significant VT recurrence 6 months after receiving a 25 Gy dose of radiation therapy.

These studies suggest that low-dose radiation therapy may alleviate arrhythmia episodes by improving myocardial cell conduction function. However, such low doses may not be sufficient for a curative effect on scar-related arrhythmias. Given the therapeutic potential of SBRT, extensive basic, translational, and clinical research is crucial for elucidating the therapeutic benefits, optimal treatment strategies, and appropriate patient populations for SBRT (58).

Currently, the guidelines for STAR treatment of arrhythmias remain unclear. The consensus on STAR was updated in 2024 (59), but it is limited to refractory arrhythmias. Strong agreement was reached on beam technique planning, dose calculation, prescription methods, and balancing the target with critical structures outside the heart. However, there is no consensus on dose limits for cardiac substructures and the required dose heterogeneity within the target area. Relatively large follow-up studies suggest that STAR appears effective and safe for patients with structural heart disease and refractory persistent VT/VF. It is associated with a significant short-term reduction in persistent VT/VF burden, but recurrences are common (60). Approximately one-third of patients undergoing STAR for refractory ventricular arrhythmias die within the first year post-procedure, with heart failure exacerbation being the primary cause of death in this population (61).

For patients with AF, one major limitation is that STAR currently focuses primarily on pulmonary vein isolation (PVI), which is a standard target in AF ablation. However, the inability to personalize the ablation set lesions based on individual substrate characteristics poses a significant challenge. Unlike conventional catheter ablation, where lesion sets can be tailored to specific patient anatomy and AF drivers, STAR’s fixed and non-adjustable approach may limit its effectiveness in cases requiring more extensive or complex ablation strategies. This limitation underscores the need for further research and development to enhance STAR’s adaptability and precision in treating AF.

Secondly, in patients with complex ventricular arrhythmias, recurrences may still occur after STAR. Although early outcomes are promising, there is a potential for scar progression in irradiated tissues, which may lead to the development of new ventricular arrhythmias.

Additionally, for VT patients with specific ion channel diseases and cardiomyopathies, such as Long QT Syndrome (LQTS) and Catecholaminergic Polymorphic Ventricular Tachycardia (CPVT), there is no evidence suggesting that STAR has a positive effect. Recent international guidelines recommend left cardiac sympathetic denervation (LCSD) as a Class I treatment for LQTS when an ICD or β-blocker therapy is ineffective (62). When the surgical risk is low, opting for cardiac sympathetic denervation can significantly reduce VT. For patients with structural heart disease (advanced heart failure) and a high surgical risk, STAR treatment targeting the stellate ganglion may be considered for those with extremely advanced and refractory heart failure.

## Advantages and prospects

Catheter ablation, as a common treatment for ventricular arrhythmias, faces a significant limitation in its operation, particularly for arrhythmias originating deep within the ventricular myocardium (midmyocardial or endocardial layers), where the heating effect of traditional catheter ablation is often restricted. This is because the thermal effect of conventional catheter ablation is difficult to penetrate deep into the myocardium, resulting in a significant disparity in treatment effectiveness compared to catheter positions at the endocardial or epicardial surfaces. However, regardless of the depth of the arrhythmia within the myocardium, STAR technology can generate effective lesion zones at the site of arrhythmia origin or its adjacent areas, thereby achieving therapeutic efficacy.

Furthermore, a major advantage of STAR technology over traditional catheter ablation procedures lies in its non-contact and non-invasive treatment approach. This method, which does not require physical contact or invasion of internal body tissues, significantly reduces the risks associated with surgery, such as infection, bleeding, or damage to surrounding tissues. Additionally, due to the minimally invasive nature of STAR technology, the postoperative recovery time for patients is greatly reduced, allowing them to resume daily life and work more quickly and substantially improving their quality of life. Therefore, STAR technology demonstrates significant potential and advantages in the treatment of deep-seated ventricular arrhythmias.
